# Molecular Biomarkers of Vascular Dysfunction in Obstructive Sleep Apnea

**DOI:** 10.1371/journal.pone.0070559

**Published:** 2013-07-29

**Authors:** Elzbieta Kaczmarek, Jessie P. Bakker, Douglas N. Clarke, Eva Csizmadia, Olivier Kocher, Aristidis Veves, Francesco Tecilazich, Christopher P. O'Donnell, Christiane Ferran, Atul Malhotra

**Affiliations:** 1 Center for Vascular Biology Research, Division of Vascular and Endovascular Surgery, Department of Surgery, Beth Israel Deaconess Medical Center, Harvard Medical School, Boston, Massachusetts, United States of America; 2 Division of Sleep Medicine, Brigham and Women's Hospital, Harvard Medical School, Boston, Massachusetts, United States of America; 3 Center for Vascular Biology Research, Department of Pathology, Beth Israel Deaconess Medical Center, Harvard Medical School, Boston, Massachusetts, United States of America; 4 Microcirculation Lab and Joslin-Beth Israel Deaconess Foot Center, Department of Surgery, Beth Israel Deaconess Medical Center, Harvard Medical School, Boston, Massachusetts, United States of America; 5 Division of Pulmonary, Allergy, and Critical Care Medicine, Department of Medicine, University of Pittsburgh, Pittsburgh, Pennsylvania, United States of America; 6 Division of Pulmonary and Critical Care Medicine, University of California San Diego, La Jolla, California, United States of America; Idaho State University, United States of America

## Abstract

Untreated and long-lasting obstructive sleep apnea (OSA) may lead to important vascular abnormalities, including endothelial cell (EC) dysfunction, hypertension, and atherosclerosis. We observed a correlation between microcirculatory reactivity and endothelium-dependent release of nitric oxide in OSA patients. Therefore, we hypothesized that OSA affects (micro)vasculature and we aimed to identify vascular gene targets of OSA that could possibly serve as reliable biomarkers of severity of the disease and possibly of vascular risk. Using quantitative RT-PCR, we evaluated gene expression in skin biopsies of OSA patients, mouse aortas from animals exposed to 4-week intermittent hypoxia (IH; rapid oscillations in oxygen desaturation and reoxygenation), and human dermal microvascular (HMVEC) and coronary artery endothelial cells (HCAEC) cultured under IH. We demonstrate a significant upregulation of endothelial nitric oxide synthase (eNOS), tumor necrosis factor-alpha-induced protein 3 (TNFAIP3; A20), hypoxia-inducible factor 1 alpha (HIF-1α?? and vascular endothelial growth factor (VEGF) expression in skin biopsies obtained from OSA patients with severe nocturnal hypoxemia (nadir saturated oxygen levels [SaO_2_]<75%) compared to mildly hypoxemic OSA patients (SaO_2_ 75%–90%) and a significant upregulation of vascular cell adhesion molecule 1 (VCAM-1) expression compared to control subjects. Gene expression profile in aortas of mice exposed to IH demonstrated a significant upregulation of eNOS and VEGF. In an *in vitro* model of OSA, IH increased expression of A20 and decreased eNOS and HIF-1α expression in HMVEC, while increased A20, VCAM-1 and HIF-1αexpression in HCAEC, indicating that EC in culture originating from distinct vascular beds respond differently to IH stress. We conclude that gene expression profiles in skin of OSA patients may correlate with disease severity and, if validated by further studies, could possibly predict vascular risk in OSA patients.

## Introduction

Symptomatic obstructive sleep apnea (OSA) is a breathing disorder that affects 6–13% of the adult Western population [1]. In addition to daytime sleepiness, OSA is implicated in the pathogenesis of cardiovascular diseases, including hypertension, coronary artery disease, congestive heart failure, stroke, cardiac arrhythmias, and sudden cardiac death. The mechanisms by which OSA affects the cardiovascular system may result from excursions in intrathoracic pressure, sympathoexcitation, and intermittent hypoxemia (IH; cycles of oxygen desaturation and re-oxygenation) [2]. Untreated OSA induces oxidative stress, inflammation, and endothelial cell (EC) dysfunction [3], which have been confirmed in animal models of OSA [4]. These abnormalities are linked to impaired activity of endothelial nitric oxide synthase (eNOS), an enzyme that generates nitric oxide (NO), and whose bioavailability is required for normal function of the endothelium [5], [6].

In the last few years, increased systemic levels of several inflammatory markers, including TNF-α, IL-6, IL-8 and ICAM-1, have been associated with OSA, suggesting that inflammation plays an important role in the pathophysiology of OSA, and possibly its vascular complications [7], [8], [9], [10], [11]. However, the role of HIF-1α, a transcription factor essential for oxygen homeostasis that is activated in response to hypoxia remains controversial in OSA studies [11], [12], [13]. Intermittent hypoxia-induced increase in HIF-1α protein levels has been suggested as an adaptive response to OSA [12], [14], [15]; however, negative effects of HIF-1α activation, such as hypertension and ischemic injury, have also been reported in animal models of OSA [16].

Although OSA is a fairly well investigated disease, the mechanistic insights into its effects on the vasculature, and specifically EC dysfunction, remain to be elucidated. Given the heavy health burden that the cardiovascular risk of OSA represents, reliable biomarkers that could estimate this risk and help define preventive and therapeutic measures are clearly needed [17]. Clinical data suggest variable cardiovascular risk in OSA populations, and indicate that both protective and deleterious pathways may be affected in OSA. Accordingly, defining the mechanisms underlying differential patient susceptibility to OSA consequences is desirable. In this study, we analyzed expression levels of select genes, chosen based on their involvement in the inflammatory/adaptive response of the vasculature to hypoxia, in skin biopsies of patients with OSA. Our aim was to identify a “gene signature” panel in the skin of OSA patients that could serve as a diagnostic and prognostic biomarker of disease severity, and ultimately to predict possible cardiovascular risk in the future, after validation in long-term clinical studies. In addition, we aimed to validate this gene signature in experimental models of OSA, using mice and *in vitro* cell cultures exposed to IH. We hypothesized that the pattern of gene regulation in mouse aorta and EC exposed to IH is also exhibited in the skin vasculature of OSA patients.

## Materials and Methods

### Participants

Non-smoking, adult subjects (median age 40 years, range 20–65; median body mass index (BMI) 42.5 kg/m^2^) were included in this study, with twelve subjects in each group: OSA patients with severe hypoxemia (apnea-hypopnea index (AHI)≥10/h, plus overnight oxygen saturation nadir <75%), OSA patients with mild hypoxemia (AHI≥10/h, oxygen saturation nadir ≥75%), and healthy controls (AHI<10/h) (Table 1). Subjects with major cardiac, respiratory, metabolic or sleep disorders other than OSA were excluded. There were no significant differences in BMI between OSA groups; however, the control group was somewhat younger than both OSA groups. All polysomnography (PSG) variables were within the normal range for the control group, with increasing AHI for the OSA groups with mild and severe hypoxemia. The study was approved by the Partners' Human Research Committee, and all subjects gave written informed consent. While some subjects participated in prior research [18], none of the findings of the present study has been previously published.

**Table 1:** Characteristics of the subjects included in the study.

	All subjects	Controls	OSA; mild hypoxemia	OSA; severe hypoxemia
	*n* = 36	*n* = 12	*n* = 12	*n* = 12
Number of males	12 (34%)	2 (17%)	5 (42%)	5 (42%)
Age (years)	40.0 (26.0)	27.5 (14.8)	51.0 (18.0)^#^	40.0 (23.5)*
BMI (kg/m^2^)	42.5 (8.3)	42.7 (8.5)	40.3 (12.2)	42.6 (16.7)
AHI (events/hour)	15.5 (31.7)	3.4 (3.7)	16.0 (8.1)^#^	52.1 (70.5)*
SaO_2_ nadir (%)	80.0 (15.8)	87.0 (7.3)	80.0 (6.0)	65.0 (13.8)* ^ †^
Percentage of time asleep with SaO_2_<90% (%)	8.9 (20.6)	1.9 (8.2)	8.6 (14.3)	44.4 (37.3)* ^ †^
Arousal index (events/hour)	18.4 (22.6)	14.3 (10.9)	23.0 (24.4)	31.3 (29.6)
Glycated hemoglobin (%)	5.6 (0.5)	5.4 (0.3)	5.7 (0.6)	5.7 (0.5)*
Total cholesterol (mg/dL)	182.5 (60.0)	186.0 (67.0)	188.0 (86.3)	179.0 (33.3)
LDL (mg/dL)	106.5 (49.0)	102.0 (51.0)	114.5 (47.0)	111.0 (45.8)
HDL (mg/dL)	46.0 (26.0)	49.0 (26.5)	45.5 (24.8)	43.0 (20.5)
Triglycerides (mg/dL)	115.5 (65.5)	127.0 (48.0)	115.5 (139.5)	99.0 (68.8)
Office systolic BP (mmHg)	116.0 (16.0)	114.0 (14.3)	116.0 (8.0)	127.0 (23.0)
Office diastolic BP (mmHg)	74.0 (12.0)	68.0 (14.5)	76.0 (8.0)	73.5 (13.8)

Gender data are presented as number (%) in each group; all other data are presented as median (interquartile range). AHI  =  apnea hypopnea index, BMI  =  body mass index, BP  =  blood pressure, HDL  =  high density lipoprotein, LDL  =  low density lipoprotein, SaO_2_  =  oxygen saturation.

* *p*≤0.05 OSA severe hypoxemia versus controls; ^#^ *p*≤0.05 OSA mild hypoxemia versus controls; ^†^ *p*≤0.05 OSA severe hypoxemia versus OSA mild hypoxemia.

### Study design

This was a cross-sectional study that consisted of a screening visit to ensure eligibility, and standard in-laboratory diagnostic polysomnography (PSG) conducted between 10 PM and 6 AM, followed by microcirculatory reactivity testing and a skin biopsy obtained after PSG completion. Subjects were asked to adhere to a low-nitrate diet for 72 h prior to admission. Subjects fasted and refrained from physical exercise from admission until test completion.

### Measurements

#### Polysomnography

PSGs were conducted and scored by blinded, registered sleep technicians according to standard criteria [16]. An apnea was scored if airflow was absent for ten seconds, and a hypopnea was scored if there was at least a 50% reduction in airflow for ten seconds or a discernable decrement in airflow for ten seconds in association with either an oxyhemoglobin desaturation of at least 3% or an arousal. An apnea-hypopnea index (AHI) was calculated based on number of apneas and hypopneas per hour of sleep.

#### Microcirculatory reactivity measurements

Microcirculatory reactivity measurements were performed between 9:30 and 11:00 AM for all subjects, following at least 30 min of seated rest in a temperature-controlled room (24–26°C). LASER Doppler flowmetry (DRT4 Monitor, Moor Instruments Ltd, UK) was used to measure skin blood flow on the ventral surface of the forearm before and after iontophoresis of acetylcholine (ACh), and before and after iontophoresis of sodium nitroprusside (SNP), using the MIC1 iontophoresis system (Moor Instruments Ltd, UK), as previously described [19]. The percentage increase in skin blood flow following ACh and SNP represents the endothelium-dependent and endothelium-independent vasodilatory response, respectively. Additional methodological details including reproducibility of the technique have been described previously [20].

#### Skin biopsies

Tissue collection was performed between 11:00 AM and 12:00 noon. Two-mm skin punch biopsies were obtained from the volar aspect of the forearm under 1% lidocaine local anesthesia. Specimens were immediately flash-frozen in liquid nitrogen and stored at −80°C.

### Mouse model of OSA

This study was approved by the Institutional Animal Care and Use Committee at the University of Pittsburgh Medical Center and complied with the American Physiological Society Guidelines for Animal Studies. Male C57BL/6J mice (20–25 g body weight, 9–12 weeks old) were purchased from the Jackson Laboratory (Bar Harbor, ME). Mice were housed in customized cages delivering IH or intermittent room air (IA; control) stimulus, as described [21]. This approach allowed mice to be maintained in their normal environment throughout the protocol. Briefly, a gas control delivery system regulated the flow of room air, N_2_, and O_2_ into the customized cages housing the mice. A series of programmable solenoids and flow regulators enabled inspired O_2_ to be varied from 20.9 to 5.0–6.0% over a 30-s period, followed by a rapid, 30-s reoxygenation to room air levels, using a burst of 100% O_2_. Hypoxic events occurred at a rate of one event per minute throughout the 12-h light period (8 AM – 8 PM). During the 12-h dark period (8 PM – 8 AM), mice were maintained in constant undisturbed room air environment. Control mice (sham exposure) were exposed to the same gas flow as IH animals, but only room air was used. Mice were exposed to 28 consecutive days of IH or IA, and then anesthetized and their aortas removed and snap frozen in liquid nitrogen.

### Cell culture and treatment

Human dermal microvascular endothelial cells (HMVEC) were obtained from Dr. Don Sanger, Beth Israel Deaconess Medical Center, Boston, MA. Human coronary artery endothelial cells (HCAEC) were purchased from Lonza (Basel, Switzerland). Cells were cultured in EGM-2 MV BulletKit medium at 37°C in a 5% CO_2_ humidified air incubator.

Confluent cells at passage 5 to 8 were used in all experiments. One ml of growth medium was added to confluent EC cultures (in 3-cm culture plates) 1 h before IH treatment. Cells (in plates without lids to obtain better exposure to gases) were placed in the IH chamber connected to the oxygen controller (Coy Laboratory Products, Inc., Grass Lake, MI, USA), and oxygen cycling was induced through controller-regulated purging of oxygen and nitrogen. Gases were delivered to the IH chamber via water bubblers placed in the tissue culture incubator to maintain adequate temperature (37°C) and humidification. Continuously monitored real-time O_2_ levels served as input to the controller feedback loop. Cells were exposed to IH for 1 and 2 h, with 9-min cycles comprised of 1.5 min ramp from 20% to 1% oxygen, 3 min 1% oxygen, 1.5 min ramp from 1 to 20% oxygen, and 3 min 20% oxygen.

### Quantitative reverse transcriptase PCR (qRT-PCR)

Total mRNA was isolated from pulverized skin biopsies or mouse aortas, using Trizol reagent (Sigma), according to the manufacturer's protocol. Total RNA from HMVEC and HCAEC was isolated using RNeasy mini kit from Qiagen (Valencia, CA, USA). cDNA was synthesized using iScript cDNA synthesis kit (Bio-Rad, Hercules, CA, USA) immediately after RNA isolation (to avoid storage-related degradation of RNA samples). Equal amounts of cDNA, an equivalent of 2.5 ng (skin biopsies and mouse aortas) or 5 ng (HCAEC and HMVEC) of RNA, were used in each reaction carried out in iTaq Fast SYBR Green Supermix with ROX (Bio-Rad, Hercules, CA, USA) using ABI 7500 Fast Real-time PCR System (Applied Biosystems, Inc., Foster City, CA, USA).

mRNA levels of eNOS, A20, VCAM-1, HIF-1α, and VEGF were evaluated using gene-specific primers (Table 2). The housekeeping genes, 28S or β-actin were used to normalize gene expression levels. The gene expression is presented as relative mRNA expression versus a control group.

**Table 2:** Sequences of primers used in qRT-PCR experiments.

GENE	ACESSION	FORWARD PRIMER	REVERSE PRIMER
H eNOS	NM_000603	GTTTGTCTGCGGCGATGTT	GCGTGAGCCCGAAAATGTC
H/M A20	NM_009397	CCTCTTCTTCGCCTGCTTTGTCC	CCCCGTCACCAAGCCGTTGTACC
H VCAM-1	NM_001078	AAGATGGTCGTGATCCTTGG	GGTGCTGCAAGTCAATGAGA
H HIF-1α	NM_001530	TGCACAGGCCACATTCACGTA	GTTCACAAATCAGCACCAAGCA
H VEGF	NM_001171623	GAGCTTCCTACAGCACAACA	GGATTTCTTGCGCTTTCGTT
H β-actin	NM_001101	GGCACCACACCTTCTACAA	AGCCTGGATAGCAACGTAC
H 28S	NM_014018	CAGTTCTCTTGGGAATCCAG	TTCAGCAAAGGAGTCAATCCAC
M eNOS	NM_008713	GTTTGTCTGCGGCGATGTT	GCGTGAGCCCGAAAATGTC
M VCAM-1	NM_011693	CTAATTCATGGTAGAATGGCTA	TGAAGTCGCATTTAAATCAGGT
M HIF-1α	NM_010431	AAACCAGCAGTTACTCATGCAA	CATGATCCAGGCTTAACAATTCCA
M VEGF	NM_001025257	CATCTTCAAGCCGTCCTGTGT	CTCCAGGGCTTCATCGTTACA
M 28S	NR_003279	ATACCGGCACGAGACCGATAGTCA	GCGGACCCCACCCGTTTACCTC

H – human, M – mouse.

### Statistical analysis

Variables exhibiting non-Gaussian distribution (human skin and mouse aortas) were transformed using log10 to satisfy normality (D'Agostino-Pearson normality test or Kolmogorov-Smirnov normality test, when n<10) and equality of variance (Levene's test). Two-group data were compared using t-test. Three-group parametric data were compared using one-way ANOVA followed by Tukey's pairwise comparisons. Three-group non-parametric results were compared globally using Kruskal-Wallis tests, followed by Dunn's pairwise comparisons. Results were presented as median or mean +/− SDEV, and were considered statistically significant when *p*<0.05 (2-sided).

## Results

### Endothelium-dependent and -independent microvascular reactivity

**Figure 1:**
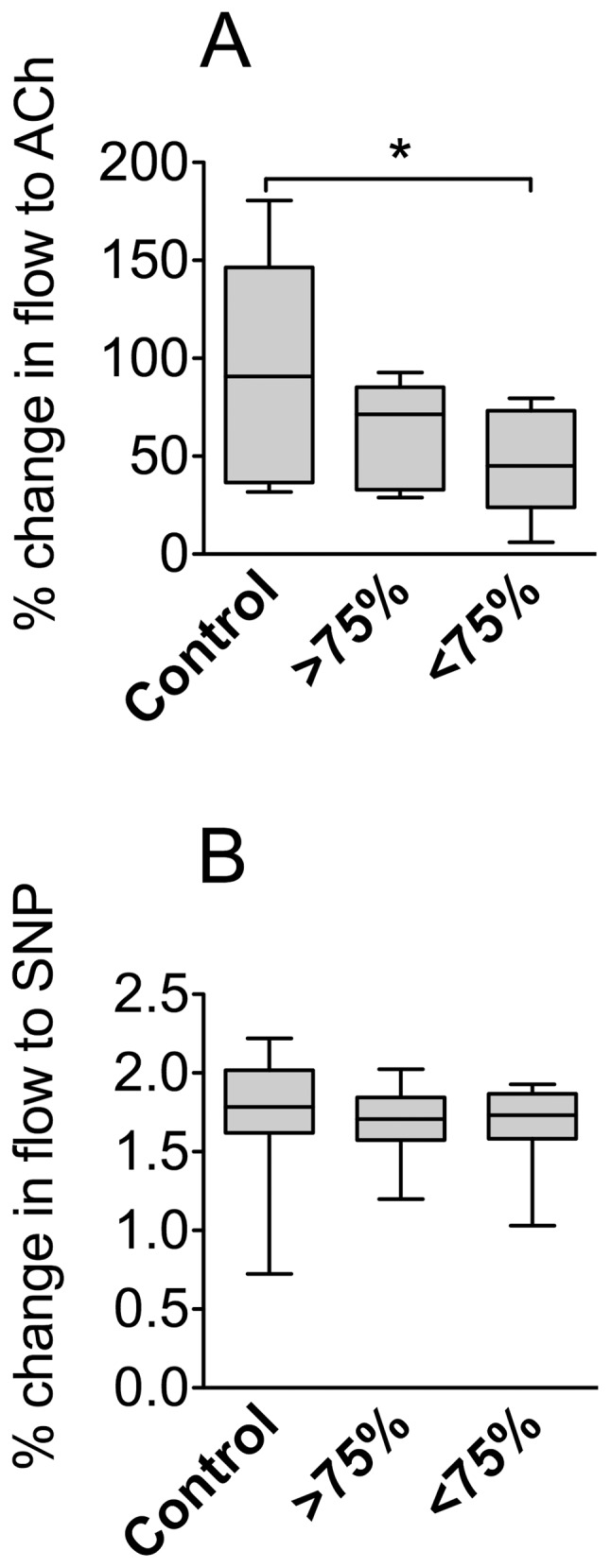
Endothelium-dependent microvascular reactivity is decreased in severely hypoxemic OSA patients. Changes in skin blood flow were measured at the forearm by scanning LASER Doppler following administration of acetylcholine (ACh) that stimulates the endothelium-dependent release of nitric oxide (**A**) or SNP that releases nitric oxide in an endothelium-independent manner (**B**). Data are presented as box-plots with medians, quartiles and minimum and maximum values; 12 subjects per group. C – control group; ≥75% – OSA group with mild hypoxemia; <75% – OSA group with severe hypoxemia.

Our results indicate that endothelium-dependent microvascular reactivity in response to ACh was significantly decreased in severely hypoxemic OSA patients compared to controls (Fig. 1A). In contrast, endothelium-independent microvascular reactivity following administration of SNP was not different across groups (Fig. 1B).

### Expression levels of select genes in skin biopsies of OSA patients

Our results demonstrating decreased microvascular reactivity in severely hypoxemic OSA patients prompted us to investigate whether OSA affects the expression level of genes involved in EC homeostasis, as well as in adaptive and inflammatory responses of the vasculature. These included EC specific NOS (eNOS) [22], [23], [24], early response anti-inflammatory gene, A20 [25], [26], pro-inflammatory adhesion molecule VCAM-1 [27], [28], and hypoxia-responsive genes, HIF-1α and VEGF [29], [30].

**Figure 2:**
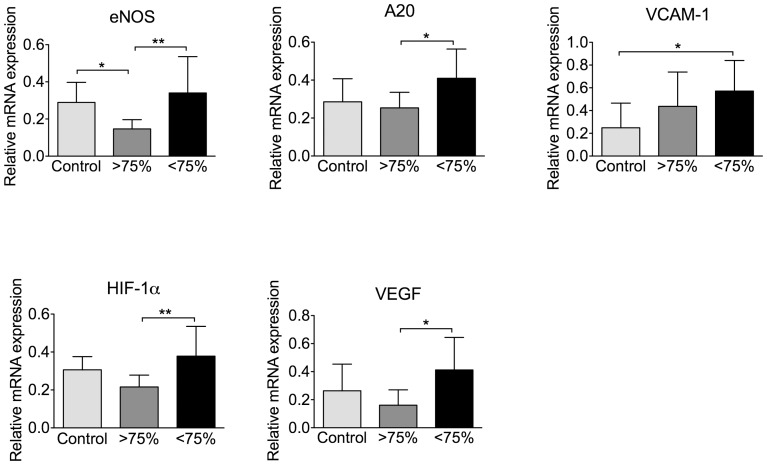
Expression of select genes in skin of OSA patients is differently regulated in severely hypoxemic (<75% blood oxygen saturation) and mildly hypoxemic (≥75% blood oxygen saturation) OSA groups. Gene expression in skin biopsies obtained from OSA patients and control subjects was analyzed by qRT-PCR using specific primers, and is presented as relative mRNA expression versus a control group. Results for each sample were normalized versus 28S. n = 10–12. Data are presented as mean +/− SDEV. * p<0.05; ** p<0.005. There is a global statistical significance for all genes in three groups of studied subjects. ≥75% – OSA group with mild hypoxemia; <75% – OSA group with severe hypoxemia.

As shown in Figure 2, expression of these genes in severely hypoxemic OSA patients was significantly increased compared to control subjects (VCAM-1) or to mildly hypoxemic OSA subjects (the other genes). Levels of eNOS were decreased in mildly hypoxemic OSA patients as compared to controls. With the exception of VCAM-1, differences in gene expression were most evident between severely and mildly hypoxemic OSA patients.

### Expression of select genes in aortas isolated from mice exposed to chronic IH

**Figure 3:**
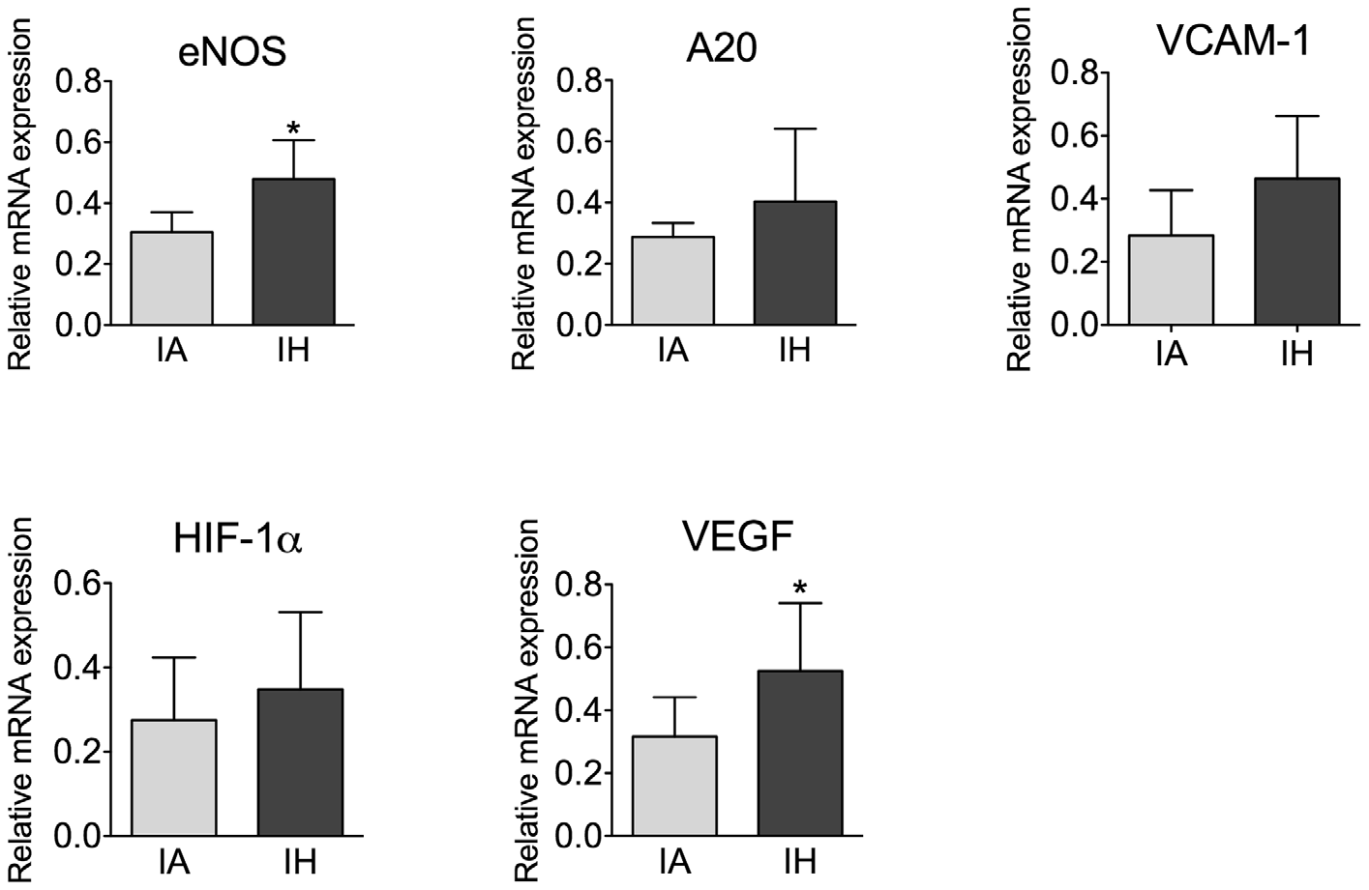
Expression of select genes in mouse aortas is affected by IH. Mice were exposed to intermittent hypoxia (IH) or intermittent air (IA) for 4 weeks. RNA was isolated from mouse aortas followed by cDNA generation and qPCR analysis. The gene expression is presented as relative mRNA expression versus a control (IA) group. Results for each sample were normalized versus 28S. Data are presented as mean +/− SDEV. n = 7–12. * p<0.05.

We investigated whether the gene expression signature we identified in the skin vasculature of OSA patients could be validated in a mouse model of OSA. We measured relative expression of the same select genes in aortas, as this vascular bed is targeted for development of accelerated atherosclerosis in OSA patients [31]. Expression levels of eNOS and VEGF mRNA were significantly upregulated in mice exposed to IH as compared to IA (Fig. 3). A similar trend, albeit not significant, was observed for A20, VCAM-1, and HIF-1α.

### Expression of select genes in HMVEC and HCAEC exposed to IH

An *in vitro* model of OSA is desirable to study the mechanisms causing vascular dysfunction of OSA/IH. We chose HMVEC in order to investigate a similar vascular bed as in our human skin biopsies, and HCAEC, as the cells preferentially used to study atherosclerosis and endothelial (dys)function.

**Figure 4:**
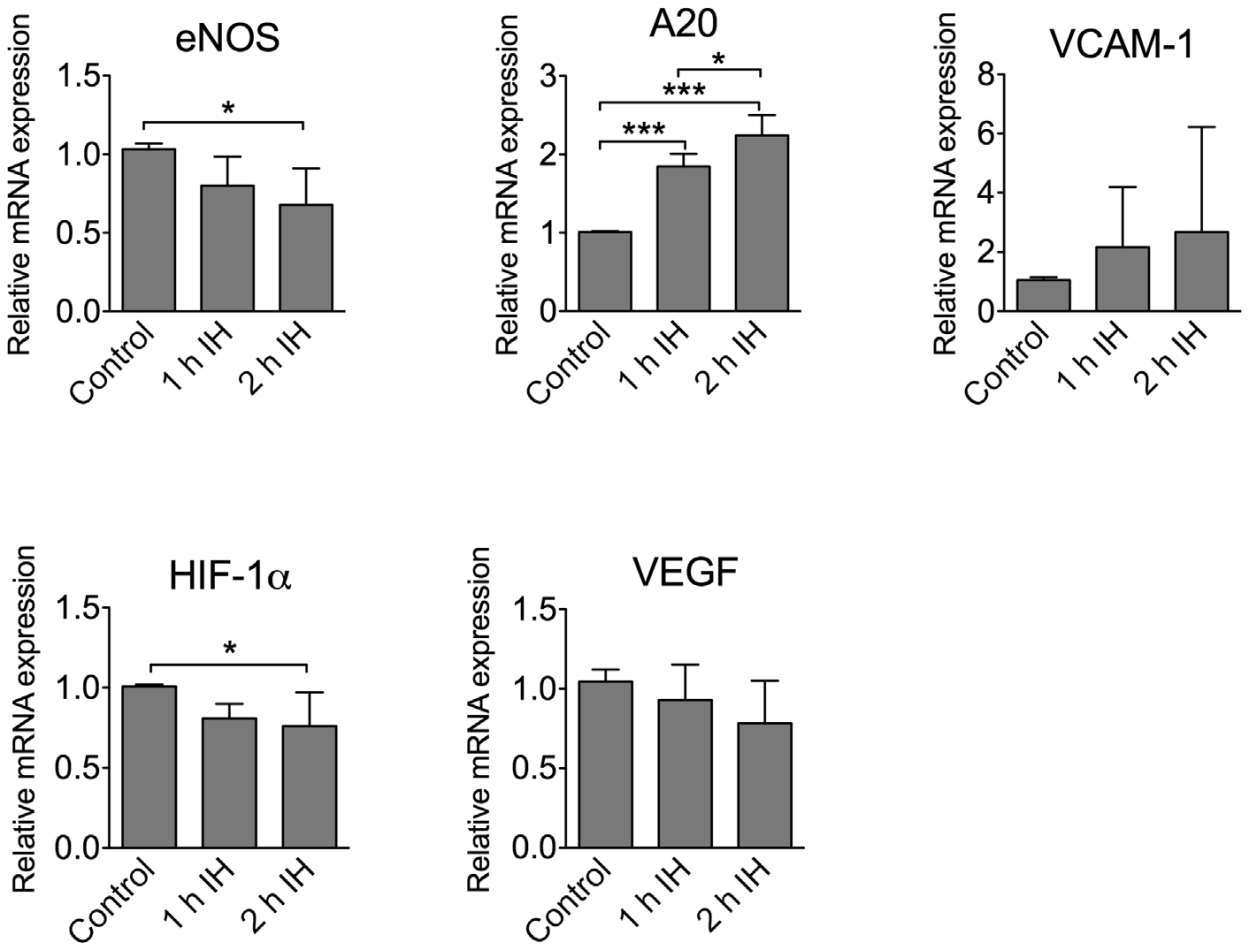
Expression of select genes in HMVEC exposed to IH. RNA was isolated from HMVEC exposed to IH, followed by cDNA generation and qRT-PCR analysis. The gene expression is presented as relative mRNA expression versus a control group. Results were obtained from at least 3 experiments and each sample was normalized versus 28S. Data are presented as mean +/− SDEV. * p<0.05; *** p<0.001.

Expression levels of eNOS and HIF-1α were significantly decreased in HMVEC after 2 h of IH (Fig. 4). We noted a similar trend (though not significant) for VEGF. In contrast, A20 levels were increased after 1 and 2 h of IH in these cells.

**Figure 5:**
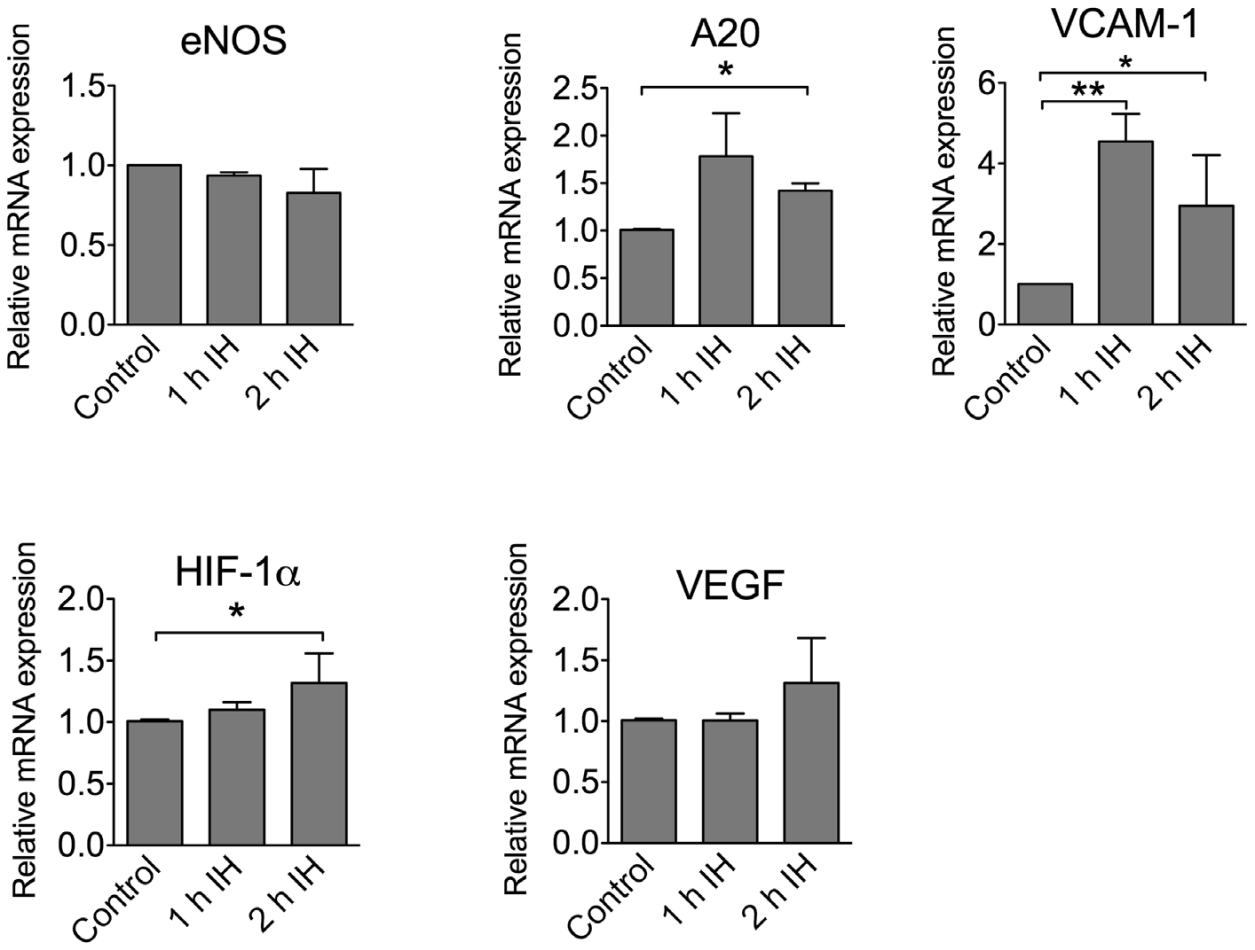
Expression of select genes in HCAEC exposed to IH. RNA was isolated from HCAEC exposed to IH, followed by cDNA generation and qRT-PCR analysis. The gene expression is presented as relative mRNA expression versus a control group. Results were obtained from at least 3 experiments and each sample was normalized versus β-actin. Data are presented as mean +/− SDEV. * p<0.05.

In HCAEC 2-h exposure to IH significantly increased expression of A20, VCAM-1, and hypoxia-responsive gene HIF-1α compared to controls (Fig. 5).

## Discussion

Identifying a “molecular signature” that could define and/or predict cardiovascular risk of OSA could be highly beneficial for diagnostic and prognostic purposes, to evaluate response to therapies, and to elucidate mechanisms involved in OSA-mediated vascular dysfunction [17]. In this study, using a minimally invasive skin biopsy method, we demonstrate, for the first time, that expression levels of several genes relevant to EC function are modulated in OSA patients in a way that correlates with disease severity, and possibly vascular risk.

The gene panel that we investigated included eNOS, whose function is crucial for EC homeostasis [22], [32], the NF-κB inhibitory gene, A20 [25], [33], the pro-inflammatory adhesion molecule VCAM-1 [27], [28], the hypoxia-responsive genes, HIF-1α and VEGF [29], [30]. Due to the experimental constraints (small sample size) our experiments were designed to study the changes at the mRNA level only, which may not correspond directly to the protein expression.

OSA patients have impaired endothelium-dependent vascular relaxation, as a result of reduced NO bioavailability caused by decreased eNOS expression and/or activity [3], [24], [33], [34], [35], [10]. Our data demonstrate that in mildly hypoxemic OSA patients, despite decreased eNOS mRNA, microvascular reactivity to acetylcholine treatment was almost not affected compared to the control group. We postulate that these patients likely produced sufficient NO to maintain proper vasoreactivity. The molecular mechanism behind reduced eNOS mRNA levels in response to mild hypoxemia still needs to be explored.

Unexpectedly, eNOS mRNA levels in severely hypoxemic OSA patients were comparable to those in controls. However, despite adequate eNOS mRNA levels, these patients showed significantly impaired microvascular reactivity, which indicates reduced eNOS activity and NO bioavailability [10]. We believe that decreased eNOS activity may result from its post-translational modification induced by OSA-triggered inflammation [8], [9], [10] that is validated here by significantly higher VCAM-1 expression in the severely hypoxemic group compared to control subjects. Future studies will verify this hypothesis, though eNOS function impairment by post-translational modifications, independently from its expression levels, was already documented in response to hypoxia and in diabetic patients [35], [36], [37], [38], [39], [40]. From a clinical standpoint, our data highlight the complexity of mechanisms regulating eNOS expression and activity in the context of severity of intermittent hypoxemia.

Our *in vivo* data demonstrate upregulation of eNOS mRNA in aortas isolated from mice exposed to chronic, 4-week IH, compared to control mice. It has been previously established that in *in vivo* model of OSA, response to IH during mice sleep time resulted in severe hypoxemia [41]. Accordingly, we are exploring whether this OSA mouse model resembles what we observed in severely hypoxemic OSA patients, i.e., that increased eNOS mRNA levels associate with severely decreased eNOS activity, resulting in vascular dysfunction; especially that these mice also demonstrate vascular inflammation [42], [43], [44].

In *an in vitro* model of IH in HMVEC eNOS mRNA levels were decreased, suggesting that even short-term exposure to IH causes changes similar to those described earlier in OSA patients and OSA *in vitro* model [6], [45], [46]. However, eNOS mRNA levels did not change in HCAEC following exposure to IH, indicating that EC from distinct vascular beds respond differently to the same hypoxic insult.

In addition to its impact on eNOS, IH, a critical component of OSA, promotes oxidative stress within the vasculature, causing vascular and systemic inflammation that culminates in vascular remodeling and atherosclerosis. Several studies reported increased levels of proinflammatory molecules in OSA patients [7], [47], [48], [49], [50], [51]. We confirmed that the systemic inflammatory response associated with OSA was also observed in severely hypoxemic patients' skin biopsies, as evaluated by increased mRNA levels of VCAM-1. Similarly, we noted some increase in VCAM-1 mRNA levels in aortas of mice exposed to IH compared to mice placed under IA, and in HCAEC exposed to IH compared to a normoxic control. Beyond supporting existing data [52], [53], these results validate our mouse and cell culture models of OSA, as they demonstrate the expected inflammatory response to hypoxic insult.

Moreover, we analyzed expression levels of the NF-κB-dependent and NF-κB inhibitory protein A20 [25], [33], [54], [55]. We have previously shown that A20 exerts protective, anti-inflammatory and anti-apoptotic functions in EC [33], [55], [56]. Our data show that A20 mRNA was significantly increased in skin biopsies of severely hypoxemic compared to mildly hypoxemic OSA patients, which indicates that the inflammatory insult associated with mild hypoxemia is not sufficient to upregulate A20 transcription. A20 mRNA levels were also increased in our *in vitro* models of OSA. Elevation of A20 in response to IH reveals the presence of an inflammatory milieu associated with chronic OSA, and is in agreement with observed upregulation of other NF-κB-dependent genes, such as VCAM-1. Alternatively, upregulation of A20 could result from hypoxia-induced increase in A20 transcription through activation of a hypoxia-response element (A/(G)CGTG) recently identified in the A20 promoter in glioblastoma cell-lines [57].

We also analyzed the expression of HIF-1α, a transcriptional regulator of oxygen homeostasis, and its downstream target VEGF [58], [59], [60], in skin biopsies of OSA patients, and in mouse aortas and EC cultures exposed to IH. Both HIF-1α and VEGF mRNA levels were higher in skin of severely hypoxemic OSA patients compared to mildly hypoxemic group. VEGF expression was also upregulated in aortas of mice exposed to IH compared to their respective controls. These findings indicate that in those tissues only a significant hypoxic insult exerts a response to hypoxia. The different effects of IH on HIF-1α mRNA levels in HMVEC and HCAEC further highlight heterogeneity among EC originating from different vascular beds, mainly in terms of their susceptibility to IH.

While many studies support the hypothesis that IH upregulates HIF-1α, some reports show no impact of OSA on HIF-1α expression [61]. Although we have not confirmed that HIF-1α mRNA levels translate into protein, we have indirect evidence that HIF-1α protein levels likely parallel mRNA levels [62]. VEGF promotes angiogenesis, a key physiologic adaptive response of tissues to hypoxia and probably IH. VEGF has also been implicated through its pro-inflammatory effects in the pathogenesis of atherosclerosis [63], [64]. Further studies are planned to evaluate whether increased VEGF in severely hypoxemic patients is truly adaptive and protective of cardiovascular events or, contrary, is a marker of increased inflammation, and hence of increased cardiovascular risk. Some studies do suggest that OSA patients free of known cardiovascular risk factors have increased circulating levels of VEGF [65], [66], [67], [68].

Likewise, we plan to evaluate whether decreased VEGF mRNA levels in patients with mild hypoxemia are indicative of increased vascular risk, or mark a lower inflammatory insult and hence lesser vascular risk. More mechanistic studies addressing these questions could now be envisioned in our mouse and cell culture models of IH, although we do recognize that the cell culture system is limited to studying EC responses, whereas gene profiling obtained from skin biopsies and whole aortas reflects the “transcriptome” of several cell types that can also modulate their expression of HIF-1α, and VEGF in response to IH.

In summary, our data demonstrate that gene expression profile in skin biopsies of OSA patients varied according to severity of hypoxemia. Even though more investigations are required to determine the contribution of these differences in mRNA levels of eNOS, VEGF, A20 and HIF-1α to the pathophysiology of OSA-induced vascular dysfunction, these genes represent potential markers distinguishing mildly from severely hypoxemic OSA patients. Since the genes we investigated are relevant to EC functions, we anticipate that their molecular signature could be useful in evaluating the cardiovascular risk in OSA patients. Further long-term studies of a larger cohort of patients are planned to validate this assumption.
